# How empathic is your healthcare practitioner? A systematic review and meta-analysis of patient surveys

**DOI:** 10.1186/s12909-017-0967-3

**Published:** 2017-08-21

**Authors:** J. Howick, L. Steinkopf, A. Ulyte, N. Roberts, K. Meissner

**Affiliations:** 1 Nuffield Department of Primary Care Health Sciences, University of Oxford, Oxford, OX2 6GG UK; 2 Institute of Medical Psychology, Faculty of Medicine, LMU Munich, 80336 Munich, Germany; 3 Faculty of Medicine, Vilnius University, 01513 Vilnius, Lithuania; 4 Bodleian Health Care Libraries, Knowledge Centre, University of Oxford, Old Road Campus Research Building, Headington, Oxford, OX3 7DQ UK; 5 Division Health Promotion, University of Applied Sciences Coburg, 95450 Coburg, Germany

**Keywords:** Empathy, Consultation, Communication, Practitioner, Expectations

## Abstract

**Background:**

A growing body of evidence suggests that healthcare practitioners who enhance how they express empathy can improve patient health, and reduce medico-legal risk. However we do not know how consistently healthcare practitioners express adequate empathy. In this study, we addressed this gap by investigating patient rankings of practitioner empathy.

**Methods:**

We conducted a systematic review and meta-analysis of studies that asked patients to rate their practitioners’ empathy using the Consultation and Relational Empathy (CARE) measure. CARE is emerging as the most common and best-validated patient rating of practitioner empathy. We searched: MEDLINE, Embase, PsycINFO, Cinahl, Science & Social Science Citation Indexes, the Cochrane Library and PubMed from database inception to March 2016. We excluded studies that did not use the CARE measure. Two reviewers independently screened titles and extracted data on average CARE scores, demographic data for patients and practitioners, and type of healthcare practitioners.

**Results:**

Sixty-four independent studies within 51 publications had sufficient data to pool. The average CARE score was 40.48 (95% CI, 39.24 to 41.72). This rank s in the bottom 5th percentile in comparison with scores collected by CARE developers. Longer consultations (*n* = 13) scored 15% higher (42.60, 95% CI 40.66 to 44.54) than shorter (*n* = 9) consultations (34.93, 95% CI 32.63 to 37.24). Studies with mostly (>50%) female practitioners (*n* = 6) showed 16% higher empathy scores (42.77, 95% CI 38.98 to 46.56) than those with mostly (>50%) male (*n* = 6) practitioners (34.84, 95% CI 30.98 to 38.71). There were statistically significant (*P =* 0.032) differences between types of providers (allied health professionals, medical students, physicians, and traditional Chinese doctors). Allied Health Professionals (*n* = 6) scored the highest (45.29, 95% CI 41.38 to 49.20), and physicians (*n* = 39) scored the lowest (39.68, 95% CI 38.29 to 41.08). Patients in Australia, the USA, and the UK reported highest empathy ratings (>43 average CARE), with lowest scores (<35 average CARE scores) in Hong Kong.

**Conclusions:**

Patient rankings of practitioner empathy are highly variable, with female practitioners expressing empathy to patients more effectively than male practitioners. The high variability of patient rating of practitioner empathy is likely to be associated with variable patient health outcomes. Limitations included frequent failure to report response rates introducing a risk of response bias. Future work is warranted to investigate ways to reduce the variability in practitioner empathy.

**Electronic supplementary material:**

The online version of this article (doi:10.1186/s12909-017-0967-3) contains supplementary material, which is available to authorized users.

## Background

A growing number of randomized trials show that when healthcare practitioners are encouraged to enhance how they express empathy, this can reduce patient pain, [1, 2] lower patient anxiety, [3] increase patient satisfaction, [4, 5] improve medication adherence, [6, 7] and ameliorate other patient health outcomes. [8–11]. For example, Chassany’s [1] empathy training intervention for general practitioners (GPs) (*n* = 180) reduced pain in osteoarthritis patients (*n* = 842) by one point on a 10-point VAS (*P* < 0.0001). These modest benefits are comparable to many pharmaceutical interventions without the adverse events. Hence some authors have recently called for efforts to encourage empathic care [12].

Supporting the view that empathic care should be encouraged, the extent to which healthcare practitioners express empathy seems to be lacking in some cases, [13–16] and it may decline with time in practice [17]. The increased burden of paperwork, which takes up a quarter of practitioner time, [18] may be a barrier to empathic care. However we do not know the prevalence of inadequate empathy. If adequate empathy is rare, then patients and practitioners would both likely benefit if practitioners reinforced how they display empathy. In this study, we aimed to address this gap by conducting a systematic review of patient ratings of practitioner empathy.

An obstacle to empathy research is that practitioner empathy is difficult to define theoretically [19, 20]. At the same time there is an emerging consensus that empathy can be operationalized as a healthcare practitioner’s ability to understand a patient’s point of view, express this understanding, and make a recommendation that reflects the shared understanding [21, 22]. More importantly for present purposes, while empathy is measured using different scales, [23, 24] only one patient-rating of practitioner empathy demonstrated evidence of reliability, [25] internal validity and consistency: CARE [25, 26]. From a patient health perspective, patient ratings of practitioner empathy are likely to be important. We therefore limited our review to studies that used the CARE measure.

### Objectives

Our primary objective was to measure the extent to which patients (of any type) report their healthcare practitioners (of any type) to be empathic. Our secondary objective was to compare differences in empathy ratings between different practitioner groups (male versus female, consultation times, different types of practitioners, and practitioners in different countries).

## Methods

### Protocol and registration

The protocol for this review was published in PROSPERO (record no. CRD42016037456). We made two changes to the protocol. In the protocol we proposed to analyze CARE scores before and after training, however there were insufficient studies to complete this analysis. We also had insufficient data to perform the proposed analyses comparing practitioners with 10 years or more experience with those who had less than 10 years experience. Neither of these changes was related to our main study aim.

### Eligibility criteria

We included any study where patients rated their practitioners’ empathy using the CARE measure. We included ratings of any practitioner including nurses, doctors, alternative practitioners, and medical students. We included studies in any language, provided that the translation of the CARE questionnaire was validated.

We excluded studies that used other measures of empathy, because only CARE has been validated. An added benefit of this approach is that it reduced heterogeneity. We excluded studies where practitioners were reported to have been trained in empathy prior to being rated by patients, since we were interested in pre-training empathy ratings. Where the publications included surveys of more than one group of practitioners the surveys were treated independently.

CARE asks patients to answer 10 questions about the consultation with their practitioner such as whether the practitioner: made the patient feel at ease, really listened and understood, showed compassion, and explained things clearly (see Additional file 1). Each question can be answered by ticking one of five options: poor, fair, good, very good, excellent, does not apply, with the lowest being given a score of ‘1’, and the highest a score of ‘5’. Hence, the maximum CARE score is 50. The developers of the CARE measure have produced normative values based on administration of their questionnaire [27]. They found that the mean CARE score was 45.75, and that 5% of CARE scores fell above 48.32, and 5% fell below 40.72.

### Information sources and search

We searched the following databases: MEDLINE (OvidSP) [1946–09/03/2016], Embase (OvidSP) [1974 to 2016 March 08], PsycINFO (OvidSP) [1967–09/03/2016], Cinahl (EBSCOHost), Science & Social Science Indexes (Web of Science, Thomson Reuters) [1945–09/03/2016], Cochrane Central Register of Controlled Trials [Issue 2 of 12, February 2016], Cochrance Database of Systematic Reviews [Issue 3 of 12, March 2016] and Database of Abstracts of Reviews of Effects [issue 2 of 4, April 2015] (via Cochrane Library, Wiley) and Pubmed (see Additional file 2 for search strategy). We also searched the Web of Science Core Collection, Scopus and Google Scholar for studies that have cited the CARE measure, [25] and any record that includes the full name of the measure (consultation and relational empathy). Additionally, we contacted authors of studies to ask whether they are aware of any additional studies.

### Data collection, extraction, and management

After piloting the extraction sheet by two authors (JH, KM), two authors (LS, AU) independently screened all titles and abstracts and extracted data. Discrepancies were resolved with discussion by a third author (JH). We extracted data about: type of practitioner, percentage female practitioners, country, average CARE score, and individual CARE scores (where available).

We assessed risk of bias within studies by measuring response rates. It was not feasible to assess risk of bias across studies, for example by conducting a funnel plot since there was no reason to suspect higher (or lower) CARE scores varying with sample size. There was insufficient data to investigate risk of bias across studies.

Statistical analyses were performed using the program Comprehensive Meta Analysis [28]. We provided the mean and 95% confidence interval of the CARE score. We contacted study authors via email to obtain missing data with respect to participants, outcomes, or summary data. Participant data were analysed as reported. We conducted preplanned subgroup analyses to assess the extent to which proportion of female practitioners, consultation duration, type of practitioner, and country played a role. To evaluate the predictive value of gender and consultation time with respect to CARE scores we performed a multivariable regression analysis, with gender and consultation time included as the independent variables, and CARE scores included as the dependent variable.

### Sensitivity and subgroup analyses

We conducted four preplanned subgroup analyses.

1. Longer (>10 min) consultations compared with shorter (≤ 10 min) consultations. This was based on average consultation times in UK general practice [29].
2. Gender: average empathy ratings of mostly (>50%) female compared with average ratings of mostly (>50%) male practitioners.
3. When there were at least three studies within the same country, we conducted a subgroup analysis with those three countries, and compared it with the complement. We chose three studies because fewer than three makes meta-analysis problematic and increases the likelihood of basing conclusions on anomalous results.
4. Types of practitioners (physicians, medical students, alternative practitioners, etc.). If there were at least three studies that measured patient ratings of specific types of practitioners, we conducted a subgroup analysis of this group, and compared it with the complement.

## Results

### Main results

**Fig. 1:**
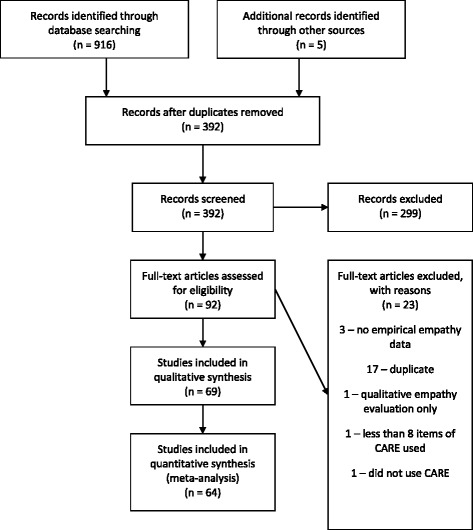
PRISMA Flow diagram

Our search yielded 392 independent records, of which 69 studies met our inclusion criteria (see Supplemental Material). Of these, 64 independent study groups (within 51 publications) had sufficient data to be included in our meta-analysis (see Table 1, Fig. 1, Additional file 3). See Additional file 4 for excluded studies.

**Table 1:** Study groups included in meta-analysis (*n* = 64 published in 51 articles)

Study	Country	Type of Providers	N Providers	% Female	N Patients	Mean (SD) consultation time (min)	Mean CARE score (SD)
Aomatsu (2014)	Japan	Physicians/Primary Care	9	N/A	272	17.2 (14.3)	38.4 (8.6)
Attar (2012)	India	Physicians/Specialists	N/A	1.00	53	N/A	29.4 (10.9)
Bikker (2005)	UK	Physicians/CAM	9	N/A	187	50.1 (14.0)	45.0 (7.0)
Bikker (2015)	UK	Nurses	17	N/A	774	13.0 (7.6)	45.9 (5.9)
Birhanu (2012)	Ethiopia	Mixed	N/A	N/A	768	6.3 (2.6)	31.3 (8.3)
Buecken (2012)	Germany	Physicians/Specialists	N/A	N/A	541	N/A	39.9 (9.1)
Chen (2015)	Hong Kong	Medical Students	158	0.39	9	15.0 (N/A)	35.8 (7.3)
Chung (2012)	South Korea	Physicians/CAM	1	0.00	143	5.0 (N/A)	38.0 (6.9)
Chung, Yip (2016)	Hong Kong	TCM practitioners	N/A	N/A	514	N/A	34.2 (8.1)
Fogarty (2013)	Australia	TCM practitioners	1	1.00	18	60.0 (N/A)	49.8 (0.6)
Fritzsche (2011a)	China	Physicians/Specialists	2	N/A	28	N/A	45.0 (5.2)
Fritzsche (2011b)	China	Physicians/Specialists	5	N/A	37	N/A	36.7 (7.7)
Fritzsche (2011c)	China	Physicians/CAM	4	N/A	31	N/A	42.9 (7.3)
Fung (2009)	Hong Kong	Physicians/Primary Care	13	N/A	228	5.7 (3.9)	31.8 (8.9)
Griffin (2014a)	UK	Physicians/Primary Care	N/A	N/A	444	N/A	39.7 (9.9)
Griffin (2014b)	UK	Nurses	N/A	N/A	444	N/A	30.4 (9.5)
Gu (2015)	Hong Kong	N/A	332	N/A	332	N/A	31.0 (9.3)
Hanzevacki (2015)	Croatia	Physicians/Primary Care	8	N/A	568	6.8 (N/A)	35.9 (4.2)
Jani (2012)	UK	Physicians/Primary Care	47	N/A	163	9.5 (4.5)	43.8 (6.9)
Johnson (2012)	UK	Mixed	21	N/A	1103	N/A	45.2 (6.2)
Johnston (2015)	UK	Mixed	17	N/A	30	N/A	39.9 (8.7)
Joice (2010)	UK	Psychotherapist	N/A	N/A	141	N/A	39.0 (8.0)
Kersten (2012)	UK	TCM practitioners	N/A	N/A	213	N/A	42.2 (6.8)
Lafreniere (2015)	US	Physicians/Specialists	44	0.57	244	41.2 (23.4)	44.6 (6.7)
LaVela (2015)	US	Physicians/Specialists	N/A	N/A	389	N/A	40.1 (9.9)
Lee (2012)	South Korea	Physicians/CAM	1	0.00	110	N/A	36.0 (8.4)
Lelorain (2015)	France	Physicians/Specialists	28	N/A	201	26.0 (14.0)	38.4 (8.9)
MacPherson (2003)	UK	TCM practitioners	N/A	N/A	135	N/A	45.5 (6.7)
Menendez (2015)	US	Physicians/Specialists	4	N/A	112	11.0 (7.0)	46.0 (6.8)
Mercer (2004)	UK	Physicians/Primary Care	N/A	N/A	10	N/A	39.2 (10.8)
Mercer (2005)	UK	Physicians/Primary Care	26	N/A	3044	N/A	40.9 (8.8)
Mercer (2008a)	UK	Physicians/Primary Care	5	0.60	323	10.0 (N/A)	42.4 (8.1)
Mercer (2008b)	UK	Physicians/Specialists	31	N/A	1582	N/A	43.8 (6.6)
Mercer (2008c)	UK	Physicians/Specialists	25	N/A	1015	N/A	43.5 (7.4)
Mercer (2011)	Hong Kong	Physicians/Primary Care	20	0.30	984	5.5 (2.9)	34.6 (8.8)
Murphy (2013a)	UK	Allied health professionals	N/A	N/A	13	N/A	43.4 (7.4)
Murphy (2013b)	UK	Physicians/Primary Care	N/A	N/A	86	N/A	43.9 (7.6)
Neumann (2007)	Germany	Physicians/Specialists	N/A	N/A	326	N/A	37.1 (11.1)
Nezenega (2013)	Ethiopia	Mixed	N/A	N/A	531	7.1 (4.4)	35.9 (8.5)
Ohm (2013)	Germany	Medical Students	30	0.73	5	N/A	41.3 (6.3)
Parrish (2016)	US	Physicians/Specialists	5	N/A	112	10.0 (5.6)	43.0 (8.0)
Place (2016a)	UK	Allied health professionals		N/A	53	N/A	45.7 (5.1)
Place (2016b)	UK	Allied health professionals		N/A	217	N/A	46.3 (5.6)
Pollak (2015)	US	Physicians/Specialists	2	N/A	21	N/A	46.0 (4.2)
Price (2006)	UK	TCM practitioners	15	N/A	52	N/A	42.4 (6.9)
Price (2008)	UK	Physicians/Primary Care	35	N/A	2550	10.2 (5.5)	43.2 (7.7)
Quaschning (2013)	Germany	Mixed	N/A	N/A	402	N/A	41.5 (7.3)
Rees (2014)	UK	Allied health professionals	N/A	N/A	225	N/A	43.1 (7.8)
Scales (2008)	US	Allied health professionals	1	N/A	411	N/A	47.6 (4.4)
Scarpellini (2014)	Brazil	Physicians/Specialists	12	N/A	12	N/A	41.4 (6.0)
Scheffer (2013a)	Germany	Medical Students	N/A	N/A	103	N/A	45.4 (5.5)
Scheffer (2013b)	Germany	Medical Students	N/A	N/A	94	N/A	41.7 (9.0)
Steinhausen (2014)	Germany	Physicians/Specialists	N/A	N/A	120	N/A	38.0 (9.8)
Tran (2012)	Australia	Physicians/Primary Care	3	N/A	38	15.0 (4.0)	43.4 (4.2)
Weiss (2015)	UK	Mixed	51	0.69	207	N/A	43.0 (7.4)
Wong (2013)	Hong Kong	Physicians/Primary Care	9	N/A	1030	7.7 (4.7)	34.4 (7.8)
Wu (2015a)	China	Physicians/Specialists	N/A	N/A	199	N/A	39.6 (8.3)
Wu (2015b)	China	Physicians/CAM	N/A	N/A	146	N/A	41.2 (8.6)
Wu (2015c)	China	Physicians/Specialists	N/A	N/A	139	N/A	38.4 (8.7)
Yu (2015a)	Hong Kong	Physicians/not specified	6	0.33	179	4.5 (2.4)	29.2 (7.4)
Yu (2015b)	Hong Kong	Physicians/Specialists	7	0.57	207	10.5 (8.6)	35.5 (8.9)
Yu (2015c)	Hong Kong	Physicians/Primary Care	14	0.50	435	7.4 (4.8)	35.7 (8.3)
Zilliacus (2011a)	Australia	Physicians/Specialists	N/A	N/A	178	N/A	41.3 (9.6)
Zilliacus (2011b)	Australia	Genetic Counselor	N/A	N/A	152	N/A	44.6 (7.8)

The 64 study groups were from 15 different countries: UK (*n* = 23), USA (*n* = 6), Hong Kong (*n* = 9), Germany (*n* = 7), Australia (*n* = 4), China (*n* = 6), Ethiopia (*n* = 2), South Korea (*n* = 2), and one study from each of Brazil, Croatia, France, India, and Japan. The types of practitioners included primary care physicians, practitioners of Traditional Chinese Medicine (TCM), medical students, allied health professionals, and other specialists.

**Fig. 2:**
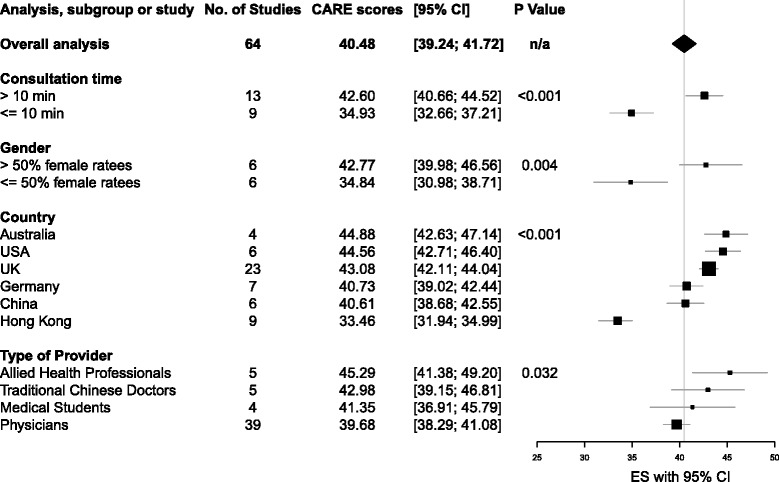
Comparison of average CARE score within subgroups

The average CARE score for the 64 study groups was 40.48 (95% CI, 39.24 to 41.72) (see Table 2, Fig. 2). Twenty-two studies reported consultation times. Longer consultations (≥10 min; *n* = 13) scored higher (42.60, 95% CI 40.69 to 44.52) than shorter (<10 min; *n* = 9) consultations (34.93, 95% CI 32.66 to 37.21). This difference of 7.67 points (15%) between longer and shorter consultations was highly significant (*P* < 0.001). Twelve studies provided data on the gender of practitioners (Table 2). Studies with predominantly female practitioners (*n* = 6) showed higher empathy scores (42.77, 95% CI 38.98 to 46.56) than those with predominantly male practitioners (*n* = 6, 34.85, 95% CI 30.98 to 38.71). This difference of 7.92 points (16%) was statistically significant (*P* = 0.004).

**Table 2:** Summary of results from subgroup analyses

Analysis	No. studies	Average CARE score (95% confidence interval)	*P*-value for difference (if applicable)
Overall	64	40.48 (39.24 to 41.72)	n/a
Longer versus shorter consultations	22		
*Longer consultations (<10 min)*	13	42.60 (40.69 to 44.52)	<0.001
*Shorter consultations (≥10 min)*	9	34.93 (32.66 to 37.21)	
Proportion of female practitioners	12		
*< 50% female practitioners*	6	34.85 (30.98 to 38.71)	0.004
*≥ 50% female practitioners*	6	42.77 (38.98 to 46.56)	
By Country	55		
UK	23	43.08 (42.11 to 44.04)	No significant difference between UK, USA, Australia, Germany and China lower than USA and Australia, Hong Kong lower than all other countries
USA	6	44.56 (42.71 to 46.40)	
Australia	4	44.88 (42.63 to 47.14)	
Germany	7	40.73 (39.02 to 42.44)	
China	6	40.61 (38.68 to 42.55)	
Hong Kong	9	33.46 (31.94 to 34.99)	
By type of provider	53		
Allied Health Professionals	5	45.29 (41.38 to 49.20)	0.032
Medical Students	4	41.35 (36.91 to 45.79)	
Physicians	39	39.68 (38.29 to 41.08)	
Traditional Chinese Doctors	5	42.98 (39.15 to 46.81)

Fifty-five study groups could be included in the pre-planned subgroup analysis by country (Table 2). Highest empathy scores were found in Australia (*n* = 4, 44.88, 95% CI 42.63 to 47.14), USA (*n* = 6, 44.56, 95% CI 42.71 to 46.40) and UK (*n* = 23, 43.07, 95% CI 42.11 to 44.04). Scores were lowest in Hong Kong (*n* = 9, 33.46, 95% CI 31.94 to 34.99). Scores in Germany (*n* = 7, 40.72, 95% CI 39.02 to 42.44) and China (*n* = 6, 40.61, 95% CI 38.68 to 42.55) were in-between. We added an exploratory analysis by country including all 64 study groups and found that scores in India (*n* = 1, 29.49, 95% CI 24.18 to 34.80) were lower than those in Hong Kong. Scores in the UK, USA and Australia were highest (See Additional file 5).

We found at least three studies each measured empathy in the following types of providers: physicians, medical students, allied health professionals, and practitioners of Traditional Chinese Medicine (Table 2). There was statistically significant heterogeneity between these (*P* = 0.032), with allied health professionals scoring the highest (*n* = 5, 45.29, 95% CI 41.38 to 49.20), and physicians scoring the lowest (*n* = 39, 39.68, 95% CI 38.29 to 41.08). We found no differences between primary care physicians, specialists, and complementary and alternative medicine (CAM) providers, (*P* = 0.386) (see Table 3).

**Table 3:** CARE scores by physician specialty

Analysis	No. studies	Average CARE score (95% confidence interval)
CAM	5	40.83 (37.78 to 43.87)
Primary Care	14	38.96 (37.14 to 40.76)
Specialists	19	40.49 (38.93 to 42.05)

A multivariable regression analysis was performed to analyze the predictive value of gender and consultation time with respect to CARE scores. Consultation duration was the only significant predictor for CARE scores (Table 4).

**Table 4:** Multivariable regression analysis, with proportion of female practitioners and consultation time as independent variables and CARE scores as dependent variable (*n* = 8)

Variable	Coefficient (ß)	Standard error	95% CI	Wald χ^2^	*P*-value
Intercept	33.40	2.82	27.87 to 38.93	11.84	<0.0001
Proportion of female practitioners	1.04	8.05	−14.74 to 16.82	0.13	0.897
Consultation duration	0.26	0.11	0.04 to 0.48	2.27	0.023

### Risk of bias

The response rate was reported in 20 of the 53 studies (38%), with the average rate being high (69%, ranging from 21% to 100%). The uncertainty about the remaining response rates entails a risk of response bias.

## Discussion

We found that patient rating of practitioner empathy is highly variable, with some practitioners being reported to express empathy much less effectively to patients than others. Female practitioners, allied health professionals, those who spend more time with patients, and practitioners from Australia, the US, and the UK seem to display empathy more effectively than other practitioners. In addition, the average care score we identified was low in comparison with normative values, falling in the lowest 5% of CARE scores measured by the developers of the questionnaire [27]. The highly variable scores we found are likely to be associated with variable patient outcomes [9–11, 30].

### Strengths and limitations

This is the first systematic review to investigate the extent to which healthcare practitioners are empathic. Another strength is that it used measures of the only validated patient-rated measure of practitioner empathy. As such, it provides a good indication of the differences between perceived empathy across gender, disciplines, and countries.

There are also several potential limitations. First, our method for measuring the difference between female and male practitioners was likely to be an underestimate. If studies with majority female practitioners resulted in greater patient-rated empathy, it is reasonable to assume that if all the practitioners were female, the difference between male and female practitioners would have been greater. In the context of this observational research we do not know whether the additional time caused female practitioners to be more empathic, or whether female practitioners’ higher empathy caused them to spend more time with patients, or whether these two factors cannot be separated. Second, response bias [26, 31, 32] could have affected the results. Patients who know they are rating their practitioners may wish to please their practitioners, [33] for example by giving them higher scores than they otherwise would [31, 32]. The lack of response rate reporting in most of the studies makes the extent of this problem unclear. Furthermore, selection bias might have influenced the results: the CARE questionnaire could be delivered in areas where the empathy of the practitioners is believed to be anomalous (either particularly high or particularly low). Next, the comparison between countries could have been influenced by the number of studies per country. Specifically, some of the countries with low scores had very few studies (Croatia had 1, Ethiopia had 2, and India had 1). Moreover in spite of validation of CARE translations, patients in different countries may have divergent prior expectations and beliefs about what it means to be an empathic practitioner. Finally, the comparison with normative values (resulting in the average score we found being in the lowest 5%) is problematic. In spite of being relatively low, the average score is still above 40. Further work needs to be done to investigate the meaning of average CARE scores.

## Conclusions

### Implications for clinical practice and clinical research

The way different healthcare practitioners express empathy to patients is low (on average) in comparison with normative scores, and highly variable. Given the likely association between practitioner empathy and patient outcomes, further research is now warranted to investigate how these findings can be used to improve patient care. Future reports of the CARE questionnaire should include all the potentially relevant factors we have identified here, especially details about response rates, and also consultation duration, gender, experience of practitioners, and other demographic details of patient raters and practitioners.

## Additional files

Additional file 1: The CARE Measure Questionnaire © Stewart W Mercer 2004**.** Actual questionnaire used within studies to measure patient perception of practitioner empathy (permission obtained). (DOCX 108 kb)

Additional file 2: Search Strategy. Search terms used to identify studies for electronic searches. (DOCX 12 kb)

Additional file 3: Studies that used the CARE measure (starred (*) studies not included in meta-analysis)**.** References to studies not included in meta-analysis because they did not meet the inclusion criteria. (DOCX 141 kb)

Additional file 4: Reasons for excluding studies identified in the search that were excluded from meta-analysis (*n* = 23)**.** Summary of justification for not including studies in meta-analysis. (DOCX 48 kb)

Additional file 5: CARE scores by country (all 64 studies included). Additional subgroup analysis by country. (DOCX 16 kb)
