# Supplementary material for: How empathic is your healthcare practitioner? A systematic review and meta-analysis of patient surveys
Source: BMC Med Educ. 2017 Aug 21;17:136. doi: 10.1186/s12909-017-0967-3 (PMC5563892; doi:10.1186/s12909-017-0967-3)
Supplement: Supplementary file 1 — The CARE Measure Questionnaire © Stewart W Mercer 2004. Actual questionnaire used within studies to measure patient perception of practitioner empathy (permission obtained). (DOCX 108 kb) [file 12909_2017_967_MOESM1_ESM.docx]

**Additional File 1.** The CARE Measure Questionnaire © Stewart W Mercer 2004. Permission has been obtained to reproduce this in a Creative Commons (CC-BY) open access publication.

| ***1.Please rate the following statements about today’s consultation*.** Please tick one box for each  statement and answer every statement. |
| --- |
| **Very Does**  **Poor Fair Good Good Excellent Not**  ***How was the doctor at*  *…*  Apply** |
| **1. Making you feel at ease*……***  *(being friendly and warm towards you,*  *treating you with respect; not cold or abrupt)* |
| **2. Letting you tell your “ story”……**  *(giving you time to fully describe your illness in*  *your own words; not interrupting or diverting you)* |
| **3. Really listening *……***  *(paying close attention to what you were sayings; not*  *looking at the notes or computer as you were talking)* |
| **4. Being interested in you as a whole person *…***  *(asking/knowing relevant details about your life,*  *your situation; not treating you as “just a number”)* |
| **5. Fully understanding your concerns*……***  *(communicating that he/she had accurately understood*  *your concerns; not overlooking or dismissing anything)* |
| **6. Showing care and compassion*….***  *(seeming genuinely concerned, connecting with you on a*  *human level; not being indifferent or “detached”)* |
| **7** **. Being Positive……**  *(having a positive approach and a positive attitude;*  *being honest but not negative about your problems)* |
| **8. Explaining things clearly*……..***  *(fully answering your questions, explaining clearly,*  *giving you adequate information; not being vague* |
| **9. Helping you to take control**……  *(exploring with you what you can do to improve your*  *health yourself; encouraging rather than “lecturing” you)* |
| **10. Making a plan of action with you *…***  *(discussing the options, involving you in decisions as*  *much as you want to be involved; not ignoring your views)* |
