# Supplementary material for: How empathic is your healthcare practitioner? A systematic review and meta-analysis of patient surveys
Source: BMC Med Educ. 2017 Aug 21;17:136. doi: 10.1186/s12909-017-0967-3 (PMC5563892; doi:10.1186/s12909-017-0967-3)
Supplement: Supplementary file 2 — Search Strategy. Search terms used to identify studies for electronic searches. (DOCX 12 kb) [file 12909_2017_967_MOESM2_ESM.docx]

**Additional File 2. Search Strategy**

Medline (Ovid) (adapted for other databases)

| 1 | "consultation and relational empathy".mp. |
| --- | --- |
| 2 | (CARE adj3 (measure* or question* or index*)).ti,ab. and empath*.mp. |
| 3 | (CARE adj3 (measure* or question* or index*)).ti,ab. and mercer.af. |
| 4 | 1 or 2 or 3 |
