# Supplementary material for: How empathic is your healthcare practitioner? A systematic review and meta-analysis of patient surveys
Source: BMC Med Educ. 2017 Aug 21;17:136. doi: 10.1186/s12909-017-0967-3 (PMC5563892; doi:10.1186/s12909-017-0967-3)
Supplement: Supplementary file 3 — Studies that used the CARE measure (starred (*) studies not included in meta-analysis). References to studies not included in meta-analysis because they did not meet the inclusion criteria. (DOCX 141 kb) [file 12909_2017_967_MOESM3_ESM.docx]

**Additional File 3. Studies that used the CARE measure (starred (*) studies not included in meta-analysis)**

Aomatsu M, Abe H, Abe K, et al. Validity and reliability of the Japanese version of the CARE measure in a general medicine outpatient setting Impact of physician empathy on migraine disability and migraineur compliance. *Family Practice* 2014; **31**(1): 118-26.

Attar HS, Chandramani S. Impact of physician empathy on migraine disability and migraineur compliance. *Annals of Indian Academy of Neurology* 2012; **15**(Suppl 1): S89-94.

***Bayne HB. Training medical students in empathic communication. *Journal for Specialists in Group Work* 2011; 36(4): 316-29.**

Bikker AP, Fitzpatrick B, Murphy D, Mercer SW. Measuring empathic, person-centred communication in primary care nurses: validity and reliability of the Consultation and Relational Empathy (CARE) Measure. *BMC Family Practice* 2015; **16**(1): 149.

Bikker AP, Mercer SW, Reilly D. A pilot prospective study on the consultation and relational empathy, patient enablement, and health changes over 12 months in patients going to the Glasgow Homoeopathic Hospital. *Journal of Alternative & Complementary Medicine* 2005; **11**(4): 591-600.

Birhanu Z, Assefa T, Woldie M, Morankar S. Predictors of perceived empathy among patients visiting primary health-care centers in central Ethiopia. *International Journal for Quality in Health Care* 2012; **24**(2): 161-8.

***Bishop FL, Yardley L, Prescott P, Cooper C, Little P, Lewith GT. Psychological Covariates of Longitudinal Changes in Back-related Disability in Patients Undergoing Acupuncture. *Clinical Journal of Pain* 2015; 31(3): 254-64.**

Buecken R, Galushko M, Golla H, et al. Patients feeling severely affected by multiple sclerosis: How do patients want to communicate about end-of-life issues? *Patient Education and Counseling* 2012; **88**(2): 318-24.

Chen JY, Chin WY, Fung CSC, Wong CKH, Tsang JPY. Assessing medical student empathy in a family medicine clinical test: validity of the CARE measure. *Medical Education Online* 2015; **20**: 27346.

Chung H, Lee H, Chang D-S, et al. Doctor's attire influences perceived empathy in the patient-doctor relationship. *Patient Education & Counseling* 2012; **89**(3): 387-91.

Chung VC, Yip BH, Yu EL, et al. Patient Perceptions of Expression of Empathy From Chinese Medicine Clinicians in a Chinese Population: A Cross-Sectional Study. *Medicine* 2016; **95**(17): e3316.

***Dodds SE, Herman PM, Sechrest L, et al. When a whole practice model is the intervention: developing fidelity evaluation components using program theory-driven science for an integrative medicine primary care clinic. *Evidence-based complementary and alternative medicine : eCAM* 2013; 2013: 652047.**

***Dossett ML, Mu L, Davis RB, et al. Patient-Provider Interactions Affect Symptoms in Gastroesophageal Reflux Disease: A Pilot Randomized, Double-Blind, Placebo-Controlled Trial. *Plos One* 2015; 10(9).**

***Eardley S. A pragmatic randomised controlled pilot study of Professional Kinesiology Practice for chronic and recurrent low back pain with initial feasibility study. *European Journal of Integrative Medicine* 2010; 2 (4): 189.**

***Eardley S, Brien S, Little P, Prescott P, Lewith G. Professional Kinesiology Practice for Chronic Low Back Pain: Single-Blind, Randomised Controlled Pilot Study. *Forschende Komplementarmedizin* 2013; 20(3): 180-8.**

Fogarty S, Smith CA, Touyz S, Madden S, Buckett G, Hay P. Patients with anorexia nervosa receiving acupuncture or acupressure, their view of the therapeutic encounter. *Complementary Therapies in Medicine* 2013; **21**(6): 675-81.

Fritzsche K, Xudong Z, Anselm K, Kern S, Wirsching M, Schaefert R. The treatment of patients with medically unexplained physical symptoms in China: A study comparing expectations and treatment satisfaction in psychosomatic medicine, biomedicine, and traditional Chinese medicine. *International Journal of Psychiatry in Medicine* 2011; **41**(3): 229-44.

Fung CSC, Hua A, Tam L, Mercer SW. Reliability and validity of the Chinese version of the CARE Measure in a primary care setting in Hong Kong. *Family Practice* 2009; **26**(5): 398-406.

Griffin SJ, Simmons RK, Prevost AT, et al. Multiple behaviour change intervention and outcomes in recently diagnosed type 2 diabetes: the ADDITION-Plus randomised controlled trial. *Diabetologia* 2014; **57**(7): 1308-19.

Gu J, Lau JTF, Wang Z, Wu AMS, Tan X. Perceived empathy of service providers mediates the association between perceived discrimination and behavioral intention to take up HIV antibody testing again among men who have sex with men. *PLoS ONE [Electronic Resource]* 2015; **10**(2): e0117376.

Hanzevacki M, Jakovina T, Bajic Z, Tomac A, Mercer S. Reliability and validity of the Croatian version of Consultation and Relational Empathy (CARE) Measure in primary care setting. *Croatian Medical Journal* 2015; **56**(1): 50-6.

Jani B, Bikker AP, Higgins M, et al. Patient centredness and the outcome of primary care consultations with patients with depression in areas of high and low socioeconomic deprivation. *British Journal of General Practice* 2012; **62**(601).

Johnson LA, Gorman C, Morse R, Firth M, Rushbrooke S. Does communication skills training make a difference to patients' experiences of consultations in oncology and palliative care services? *European Journal of Cancer Care* 2013; **22**(2): 202-9.

Johnston B, Pringle J, Gaffney M, Narayanasamy M, McGuire M, Buchanan D. The dignified approach to care: a pilot study using the patient dignity question as an intervention to enhance dignity and person-centred care for people with palliative care needs in the acute hospital setting. *BMC Palliative Care* 2015; **14**: 9.

Joice A, Mercer SW. An evaluation of the impact of a large group psycho-education programme (Stress Control) on patient outcome: Does empathy make a difference? *the Cognitive Behaviour Therapist* 2010; **3**(1): 1-17.

Kersten P, White PJ, Tennant A. The consultation and relational empathy measure: an investigation of its scaling structure. *Disability & Rehabilitation* 2012; **34**(6): 503-9.

Lafreniere JP, Rios R, Packer H, Ghazarian S, Wright SM, Levine RB. Burned Out at the Bedside: Patient Perceptions of Physician Burnout in an Internal Medicine Resident Continuity Clinic. *Journal of General Internal Medicine* 2016; **31**(2): 203-8.

***Landon A, Neilens H, GerickeC, George J, Freeman R. Patient satisfaction with telephone review compared to outpatient review following urogynaecological surgery: A pilot randomised controlled trial. *BJOG: An International Journal of Obstetrics and Gynaecology* 2014; 121 (7): e10.**

LaVela SL, Heinemann AW, Etingen B, Miskovic A, Locatelli SM, Chen D. Relational empathy and holistic care in persons with spinal cord injuries. *The journal of spinal cord medicine* 2015: 1-20.

Lee S-H, Chang D-S, Kang OS, et al. Do not judge according to appearance: patients' preference of a doctor's face does not influence their assessment of the patient-doctor relationship. *Acupuncture in Medicine* 2012; **30**(4): 261-5.

Lelorain S, Bredart A, Dolbeault S, et al. How does a physician's accurate understanding of a cancer patient's unmet needs contribute to patient perception of physician empathy? *Patient Education and Counseling* 2015; **98**(6): 734-41.

MacPherson H, Mercer SW, Scullion T, Thomas KJ. Empathy, enablement, and outcome: an exploratory study on acupuncture patients' perceptions. *Journal of Alternative & Complementary Medicine* 2003; **9**(6): 869-76.

Menendez ME, Chen NC, Mudgal CS, Jupiter JB, Ring D. Physician Empathy as a Driver of Hand Surgery Patient Satisfaction. *Journal of Hand Surgery - American Volume* 2015; **40**(9): 1860-5.e2.

Mercer SW, Fung CSC, Chan FWK, Wong FYY, Wong SYS, Murphy D. The Chinese-version of the CARE measure reliably differentiates between doctors in primary care: a cross-sectional study in Hong Kong. *BMC Family Practice* 2011; **12**: 43.

Mercer SW, Hatch DJ, Murray A, Murphy DJ, Eva KW. Capturing patients' views on communication with anaesthetists: The CARE Measure. *Clinical Governance* 2008; **13**(2): 128-37.

Mercer SW, Maxwell M, Heaney D, Watt GC. The consultation and relational empathy (CARE) measure: development and preliminary validation and reliability of an empathy-based consultation process measure. *Family Practice* 2004; **21**(6): 699-705.

Mercer SW, McConnachie A, Maxwell M, Heaney D, Watt GCM. Relevance and practical use of the Consultation and Relational Empathy (CARE) Measure in general practice. *Family Practice* 2005; **22**(3): 328-34.

Mercer SW, Murphy DJ. Validity and reliability of the CARE Measure in secondary care. *Clinical Governance* 2008; **13**(4): 269-83.

Mercer SW, Neumann M, Wirtz M, Fitzpatrick B, Vojt G. General practitioner empathy, patient enablement, and patient-reported outcomes in primary care in an area of high socio-economic deprivation in Scotland--a pilot prospective study using structural equation modeling. *Patient Education & Counseling* 2008; **73**(2): 240-5.

***Munshi S, McGrady E, Young S. Piloting the care revalidation tool in obstetric anaesthesia. *International Journal of Obstetric Anesthesia* 2013; 22: S52.**

***Murphy DJ, Bruce DA, Mercer SW, Eva KW. The reliability of workplace-based assessment in postgraduate medical education and training: a national evaluation in general practice in the United Kingdom. *Advances in Health Sciences Education* 2009; 14(2): 219-32.**

Murphy J, Mercer SW, Duncan EAS. A pilot study to explore the feasibility, validity and reliability of a visual version of the CARE Measure. *International Journal of Therapy and Rehabilitation* 2013; **20**(9): 460-5.

Neumann M, Wirtz M, Bollschweiler E, et al. Determinants and patient-reported long-term outcomes of physician empathy in oncology: a structural equation modelling approach. *Patient Education & Counseling* 2007; **69**(1-3): 63-75.

Nezenega ZS, Gacho YHM, Tafere TE. Patient satisfaction on tuberculosis treatment service and adherence to treatment in public health facilities of Sidama zone, South Ethiopia. *BMC Health Services Research* 2013; **13**: 110.

Ohm F, Vogel D, Sehner S, Wijnen-Meijer M, Harendza S. Details acquired from medical history and patients' experience of empathy--two sides of the same coin. *BMC Medical Education* 2013; **13**: 67.

Parrish RC, 2nd, Menendez ME, Mudgal CS, Jupiter JB, Chen NC, Ring D. Patient Satisfaction and its Relation to Perceived Visit Duration With a Hand Surgeon. *Journal of Hand Surgery - American Volume* 2016; **41**(2): 257-62.e4.

***Pick V, Halstenberg K, Demel A, et al. Staff and parents are discriminators for outcomes in neonatal intensive care units. *Acta Paediatrica* 2014; 103(11): E475-E83.**

Place MA, Murphy J, Duncan EA, Reid JM, Mercer SW. A preliminary evaluation of the Visual CARE Measure for use by Allied Health Professionals with children and their parents. *Journal of Child Health Care* 2016; **20**(1): 55-67.

Pollak KI, Jones J, Lum HD, et al. Patient and caregiver opinions of motivational interviewing techniques in role-played palliative care conversations: A pilot study. *Journal of Pain and Symptom Management* 2015; **50**(1): 91-8.

Price R, Spencer J, Walker J. Does the presence of medical students affect quality in general practice consultations? *Medical Education* 2008; **42**(4): 374-81.

Price S, Mercer SW, MacPherson H. Practitioner empathy, patient enablement and health outcomes: a prospective study of acupuncture patients. *Patient Education & Counseling* 2006; **63**(1-2): 239-45.

Quaschning K, Korner M, Wirtz M. Analyzing the effects of shared decision-making, empathy and team interaction on patient satisfaction and treatment acceptance in medical rehabilitation using a structural equation modeling approach. *Patient Education and Counseling* 2013; **91**(2): 167-75.

***Rakel D, Barrett B, Zhang Z, et al. Perception of empathy in the therapeutic encounter: effects on the common cold. *Patient Education & Counseling* 2011; 85(3): 390-7.**

Rees EL, Thomas E, Hill JC. Practitioner empathy and musculoskeletal patient outcomes in primary care. *Rheumatology (United Kingdom)* 2014; **53**: i79-i80.

***Ricci D, Chewning B, Peters J, Desai K. Secret shopper perceptions of pharmacist empathy during counseling after purchase of emergency contraceptive or ibuprofen in community pharmacies. *Journal of the American Pharmacists Association* 2015; 55 (2): e143.**

***Riess H, Kelley JM, Bailey R, Konowitz PM, Gray ST. Improving Empathy and Relational Skills in Otolaryngology Residents: A Pilot Study. *Otolaryngology-Head and Neck Surgery* 2011; 144(1): 120-2.**

***Riess H, Kelley JM, Bailey RW, Dunn EJ, Phillips M. Empathy training for resident physicians: a randomized controlled trial of a neuroscience-informed curriculum. *Journal of General Internal Medicine* 2012; 27(10): 1280-6.**

Scales R, Hartlein E, Manuel JK, et al. Assessment of motivational interviewing proficiency in cardiac rehabilitation. *Journal of Cardiopulmonary Rehabilitation and Prevention* 2008; **28 (4)**: 275.

Scarpellini GR, Capellato G, Rizzatti FG, Da Silva GA, Baddini-Martinez JA. CARE scale of empaty: Translation to the Portuguese spoken in Brazil and initial validation results. [Portuguese] Escala CARE de empatia: Traducao para o Portugues falado no Brasil e resultados iniciais de validacao. *Medicina (Brazil)* 2014; **47**(1): 51-8.

Scheffer C, Tauschel D, Neumann M, Lutz G, Valk-Draad M, Edelhauser F. Active student participation may enhance patient centeredness: patients' assessments of the clinical education ward for integrative medicine. *Evidence-Based Complementary & Alternative Medicine: eCAM* 2013; **2013**: 743832.

Steinhausen S, Ommen O, Thum S, et al. Physician empathy and subjective evaluation of medical treatment outcome in trauma surgery patients. *Patient Education & Counseling* 2014; **95**(1): 53-60.

***Thompson TDB, Weiss M. Homeopathy - What are the active ingredients? An exploratory study using the UK Medical Research Council's framework for the evaluation of complex interventions. *BMC Complementary and Alternative Medicine* 2006; 6.**

Tran PD, Laurence JM, Weston KM, McLennan PL. The effect of parallel consulting on the quality of consultations in regional general practice. *Education for Primary Care* 2012; **23**(3): 153-7.

***Turner P, Barnard ML, Harrington S. Consultation quality in the diabetes clinic. *Diabetic Medicine* 2009; 26: 126.**

Weiss MC, Platt J, Riley R, et al. Medication decision making and patient outcomes in GP, nurse and pharmacist prescriber consultations. *Primary Health Care Research and Development* 2015; **16**(5): 513-27.

***Wijnen-Meijer M, ten Cate O, van der Schaaf M, Burgers C, Borleffs J, Harendza S. Vertically integrated medical education and the readiness for practice of graduates. *Bmc Medical Education* 2015; 15.**

Wong CKM, Yip BHK, Mercer S, et al. Effect of facemasks on empathy and relational continuity: a randomised controlled trial in primary care. *BMC Family Practice* 2013; **14**: 200.

Wu H, Zhao X, Fritzsche K, et al. Quality of doctor-patient relationship in patients with high somatic symptom severity in China. *Complementary Therapies in Medicine* 2015; **23**(1): 23-31.

Yu FSK, Yip BHK, Kung K, et al. The Association of Types of Training and Practice Settings with Doctors' Empathy and Patient Enablement among Patients with Chronic Illness in Hong Kong. *PLoS ONE [Electronic Resource]* 2015; **10**(12): e0144492.

Zilliacus EM, Meiser B, Lobb EA, et al. Are videoconferenced consultations as effective as face-to-face consultations for hereditary breast and ovarian cancer genetic counseling? *Genetics in Medicine* 2011; **13**(11): 933-41.
