# Supplementary material for: How empathic is your healthcare practitioner? A systematic review and meta-analysis of patient surveys
Source: BMC Med Educ. 2017 Aug 21;17:136. doi: 10.1186/s12909-017-0967-3 (PMC5563892; doi:10.1186/s12909-017-0967-3)
Supplement: Supplementary file 4 — Reasons for excluding studies identified in the search that were excluded from meta-analysis (n = 23). Summary of justification for not including studies in meta-analysis. (DOCX 48 kb) [file 12909_2017_967_MOESM4_ESM.docx]

**Additional File 4. Reasons for excluding studies from meta-analysis (n=23) that were identified in the search**

1. No questionnaire responses (study protocol or only theoretical considerations) (n=3)[[1-3](#_ENREF_1)]

2. Subsequent (not first) report of the same study sample (n=17)[[4-20](#_ENREF_4)]

3. Qualitative empathy evaluation only (n=1)[[21](#_ENREF_21)]

4. Incomplete (fewer than 8 items of CARE used (n=1)[[22](#_ENREF_22)]

5. Did not use CARE (used other measure) (n=1)[[23](#_ENREF_23)]

**References**

1. Barrett B, Rakel D, Chewning B, Marchand L, Rabago D, Brown R, Scheder J, Schmidt R, Gern JE, Bone K *et al*: **Rationale and methods for a trial assessing placebo, echinacea, and doctor-patient interaction in the common cold**. *Explore-the Journal of Science and Healing* 2007, **3**(6):561-572.

2. Bikker AP, Mercer SW, Cotton P: **Connecting, Assessing, Responding and Empowering (CARE): A universal approach to person-centred, empathic healthcare encounters**. *Education for Primary Care* 2012, **23**(6):454-457.

3. Neumann M, Wirtz M, Ommen O, Mercer SW, Ernstmann N, Pfaff H: **Psychometric evaluation of the 'consultation and relational empathy'(care) measure in cancer care**. *Psycho-Oncology* 2009, **18**:S88.

4. Birhanu Z, Assefa T, Woldie M, Morankar S: **Determinants of satisfaction with health care provider interactions at health centres in central Ethiopia: a cross sectional study**. *BMC Health Serv Res* 2010, **10**:78.

5. Birhanu Z, Woldie MK, Assefa T, Morankar S: **Determinants of patient enablement at primary health care centres in central Ethiopia: A cross-sectional study**. *African Journal of Primary Health Care and Family Medicine* 2011, **3**(1).

6. Dambha-Miller H, Cooper AJM, Simmons RK, Kinmonth AL, Griffin SJ: **Patient-centred care, health behaviours and cardiovascular risk factor levels in people with recently diagnosed type 2 diabetes: 5-year follow-up of the ADDITION-Plus trial cohort**. *Bmj Open* 2016, **6**(1).

7. Mercer SW, Howie JGR: **CQI-2--a new measure of holistic interpersonal care in primary care consultations**. *British Journal of General Practice* 2006, **56**(525):262-268.

8. Mercer SW, Fitzpatrick B, Gourlay G, Vojt G, McConnachie A, Watt GCM: **More time for complex consultations in a high-deprivation practice is associated with increased patient enablement**. *British Journal of General Practice* 2007, **57**(545):960-966.

9. Mercer SW, Jani BD, Maxwell M, Wong SYS, Watt GCM: **Patient enablement requires physician empathy: a cross-sectional study of general practice consultations in areas of high and low socioeconomic deprivation in Scotland**. *BMC Family Practice* 2012, **13**:6.

10. Mercer SW, Watt GCM: **The inverse care law: Clinical primary care encounters in deprived and affluent areas of Scotland**. *Annals of Family Medicine* 2007, **5**(6):503-510.

11. Neumann M, Bensing J, Wirtz M, Wubker A, Scheffer C, Tauschel D, Edelhauser F, Ernstmann N, Pfaff H: **The impact of financial incentives on physician empathy: a study from the perspective of patients with private and statutory health insurance**. *Patient Education & Counseling* 2011, **84**(2):208-216.

12. Neumann M, Wirtz M, Bollschweiler E, Warm M, Wolf J, Pfaff H: **[Psychometric evaluation of the German version of the "Consultation and Relational Empathy" (CARE) measure at the example of cancer patients]**. *Psychotherapie, Psychosomatik, Medizinische Psychologie* 2008, **58**(1):5-15.

13. Neumann M, Wirtz M, Ernstmann N, Ommen O, Langler A, Edelhauser F, Scheffer C, Tauschel D, Pfaff H: **Identifying and predicting subgroups of information needs among cancer patients: an initial study using latent class analysis**. *Supportive care in cancer : official journal of the Multinational Association of Supportive Care in Cancer* 2011, **19**(8):1197-1209.

14. Rakel DP, Hoeft TJ, Barrett BP, Chewning BA, Craig BM, Niu M: **Practitioner empathy and the duration of the common cold**. *Family Medicine* 2009, **41**(7):494-501.

15. Wirtz M, Boecker M, Forkmann T, Neumann M: **Evaluation of the "Consultation and Relational Empathy" (CARE) measure by means of Rasch-analysis at the example of cancer patients**. *Patient Education & Counseling* 2011, **82**(3):298-306.

16. Lelorain S, Bredart A, Dolbeault S, Cano A, Bonnaud-Antignac A, Cousson-Gelie F, Sultan S: **How can we explain physician accuracy in assessing patient distress? A multilevel analysis in patients with advanced cancer**. *Patient Educ Couns* 2014, **94**(3):322-327.

17. Mercer SW: **Practitioner empathy, patient enablement and health outcomes of patients attending the Glasgow Homoeopathic Hospital: A retrospective and prospective comparison**. *Wiener Medizinische Wochenschrift* 2005, **155**(21-22):498-501.

18. Steinhausen S, Ommen O, Antoine S-L, Koehler T, Pfaff H, Neugebauer E: **Short- and long-term subjective medical treatment outcome of trauma surgery patients: the importance of physician empathy**. *Patient preference & adherence* 2014, **8**:1239-1253.

19. Chung V, Yip B, Yu E, Liu S, Sit R, Leung A, Wu J, Wong S: **Patient perceived expression of empathy from chinese medicine clinicians in Hong Kong: Does practice modality make a difference?** *Integrative Medicine Research* 2015, **1)**:17.

20. La Vela SL, Heinemann AW, Etingen B, De Mark Neumann H, Miskovic A, Locatelli SM, Chen D: **Perceptions of patient-centered care in individuals with spinal cord injuries/disorders**. *Journal of Spinal Cord Medicine* 2014, **37 (4)**:462.

21. Fung CSC, Mercer SW: **A qualitative study of patients' views on quality of primary care consultations in Hong Kong and comparison with the UK CARE Measure**. *BMC Family Practice* 2009, **10**:10.

22. Ruck JD, Shah VN, Runion AM, Snell-Bergeon JK: **Factors affecting satisfaction with providers among adults with type 1 diabetes: A web-based survey**. *Diabetes* 2015, **64**:A352.

23. Sylvia LG, Hay A, Ostacher MJ, Miklowitz DJ, Nierenberg AA, Thase ME, Sachs GS, Deckersbach T, Perlis RH: **Association between therapeutic alliance, care satisfaction, and pharmacological adherence in bipolar disorder**. *Journal of Clinical Psychopharmacology* 2013, **33**(3):343-350.
