# Supplementary material for: How empathic is your healthcare practitioner? A systematic review and meta-analysis of patient surveys
Source: BMC Med Educ. 2017 Aug 21;17:136. doi: 10.1186/s12909-017-0967-3 (PMC5563892; doi:10.1186/s12909-017-0967-3)
Supplement: Supplementary file 5 — CARE scores by country (all 64 studies included). Additional subgroup analysis by country. (DOCX 16 kb) [file 12909_2017_967_MOESM5_ESM.docx]

**Additional File 5. CARE scores by country (all 64 studies included)**

| **Analysis** | **No. studies** | **Average CARE score (95% confidence interval)** |
| --- | --- | --- |
| Australia | 4 | 44.88 (42.56 to 47.17) |
| USA | 6 | 44.55 (42.69 to 46.43) |
| UK | 23 | 43.07 (42.10 to 44.05) |
| Brazil | 1 | 41.41 (35.85 to 46.97) |
| Germany | 7 | 40.72 (38.99 to 42.60) |
| China | 6 | 40.61 (38.66 to 42.57) |
| Japan | 1 | 38.41 (33.87 to 42.95) |
| France | 1 | 38.40 (33.81 to 42.99) |
| South Korea | 2 | 37.01 (33.69 to 40.33) |
| Croatia | 1 | 35.90 (31.46 to 40.34) |
| Ethiopia | 2 | 33.60 (30.44 to 36.76) |
| Hong Kong | 9 | 33.46 (31.92 to 35.01) |
| India | 1 | 29.49 (24.18 to 34.80) |
